# Association between serum α‐Klotho levels and severity of periodontitis in a representative U.S. population

**DOI:** 10.1002/jper.70080

**Published:** 2026-02-28

**Authors:** Hamoun Sabri, Sahar Baniameri, Pranav Kataria, Hend Abulatifa, Parham Hazrati, Hom‐Lay Wang, Muhammad H. A. Saleh

**Affiliations:** ^1^ Department of Periodontics and Oral Medicine University of Michigan School of Dentistry Ann Arbor Michigan USA; ^2^ Center for Clinical Research and Evidence Synthesis in Oral Tissue Regeneration (CRITERION) Ann Arbor Michigan USA; ^3^ Department of Periodontics University of Connecticut Health Farmington Connecticut USA

**Keywords:** aging, cross‐sectional study, NHANES, periodontitis, systemic disease, α‐Klotho

## Abstract

**Background:**

Periodontitis, a chronic inflammatory disease, is linked to systemic conditions such as cardiovascular and kidney disease. Serum α‐Klotho, an anti‐aging protein with anti‐inflammatory properties, has been associated with systemic diseases, but its role in periodontitis is unclear. This study evaluated the relationship between serum α‐Klotho levels and periodontitis severity while accounting for confounders.

**Methods:**

In this cross‐sectional study, data from 961 participants in the National Health and Nutrition Examination Survey (NHANES) database were analyzed. Periodontitis was classified into stages (I–IV) and grades (A–C) using the ACES (Application of the 2018 periodontal status Classification to Epidemiological Survey data) guidelines. Serum α‐Klotho levels were measured via enzyme‐linked immunosorbent assay (ELISA). Ordinal logistic regression assessed associations between α‐Klotho levels and periodontitis, adjusting for confounders such as age, smoking, comorbidities, and oral hygiene. The number of lost teeth was analyzed as a secondary outcome.

**Results:**

In both adjusted and unadjusted regression models, no significant association was found between α‐Klotho levels and periodontitis. Particularly, adjusted models revealed no significant association between α‐Klotho levels and periodontitis stage (OR = 1.0001, *p* = 0.547, 95% CI: 0.9997–1.0006) or grade (OR = 0.9996, *p* = 0.144, 95% CI: 0.9991–1.0001). Age, smoking, and comorbidities significantly predicted severity. Despite a weak negative correlation between α‐Klotho and tooth loss (*r* = –0.07, *p* = 0.023), this association was no longer significant after adjustment.

**Conclusion:**

No significant association was found between serum α‐Klotho levels and periodontitis severity. Age, smoking, and comorbidities were key predictors, highlighting the multifactorial nature of periodontitis. Further longitudinal and mechanistic studies are needed to clarify whether α‐Klotho has a value as a biomarker of periodontal inflammation or disease progression.

## Plain language summary

α‐Klotho is an anti‐aging protein known for its protective role against oxidative stress, inflammation, and tissue destruction, processes critically involved in periodontitis pathogenesis. Although α‐Klotho levels have been associated with systemic aging‐related diseases, its relationship with periodontal disease severity remains unclear, with limited and conflicting existing evidence. This study is the first to comprehensively assess the association between serum α‐Klotho levels and periodontitis severity using the latest 2018 ACES (Application of the 2018 Classification to Epidemiological Survey Data) criteria, and applying statistical adjustments in a representative U.S. population derived from three National Health and Nutrition Examination Survey (NHANES) cycles. Unlike previous studies, we carefully accounted for key confounding factors such as demographic variables, smoking, systemic comorbidities, and oral hygiene behaviors to provide clear insights into whether α‐Klotho independently relates to periodontal disease severity and tooth loss. Despite a plausible biological rationale, we found no independent significant association between serum α‐Klotho and periodontitis severity after adjusting for relevant confounders. Clinicians should thus continue to focus on established clinical factors such as age, smoking status, systemic diseases, and socioeconomic determinants when assessing periodontitis risk and progression. Future research, particularly longitudinal studies, is necessary before considering α‐Klotho as a potential biomarker or therapeutic target in periodontal clinical practice.

## 1 INTRODUCTION

Increasing evidence suggests that periodontitis is not only a chronic inflammatory disease but may also act as a risk factor for several systemic conditions.^1^, ^2^ These include diabetes, cardiovascular disease (CVD), Alzheimer's disease, chronic kidney disease (CKD), respiratory diseases, cancer, metabolic syndrome, osteoporosis, and arthritis, among others.^3^, ^4^ This relationship is mediated by a direct and indirect pathway.^5^ The direct effect is mediated through the translocation of pathogens from dental plaque into the bloodstream, leading to bacteremia, while the indirect action is facilitated by microbial plaque inducing a host immune response, resulting in persistent inflammation.^6^

On a molecular level, periodontitis impacts immune cell function by upregulating pro‐inflammatory cytokines, resulting in dysregulated lipid metabolism and elevated oxidative stress.^7^ One such biomarker of interest is serum α‐Klotho, a protein initially identified in renal tubular cells. This anti‐aging suppressor gene encoding α‐Klotho was first discovered by Masuda et al. in 1997.^8^ Their findings demonstrated that mutations in the α‐Klotho gene in mice resulted in stunted growth, organ atrophy, hyperphosphatemia, hypercalcemia, vascular calcification, cardiac hypertrophy, multi‐organ fibrosis, and significantly shortened lifespan.^8^ Conversely, overexpression of α‐Klotho in mice extended their lifespan by up to 30%.^9^

In humans, the *Klotho* gene is located on chromosome 13q13.1 and is most abundantly expressed in the kidneys. A paralog of this gene, *β‐Klotho*, resides on chromosome 4 and is predominantly expressed in adipose tissue but is also found in skeletal tissue, the aorta, and the heart. Both α‐Klotho and β‐Klotho encode two forms of proteins: membrane‐bound and secreted.^10^ Throughout this article, the focus is solely on α‐Klotho.

Serum levels of α‐Klotho in humans decline significantly after the age of 40, correlating with the onset of several age‐related diseases, such as cancer, hypertension, and kidney disease.^10^, ^11^ Longitudinal studies have also shown a statistically significant relationship between advancing age and the incidence of periodontal disease, with an odds ratio of 10.4 for people aged 36–50.^12^ The mechanisms underlying the effects of α‐Klotho are multifaceted. Broadly, its actions include inhibition of nuclear factor‐kappa B (NF‐κB) and transforming growth factor‐β (TGF‐β), both of which are key mediators of inflammation, fibrosis, and age‐related diseases.^13^ The NF‐κB pathway is also activated by oral microbial pathogens through the toll‐like receptors and contributes to the inflammation seen in periodontitis.^14^ Additionally, ⍺‐Klotho suppresses the NLRP3 inflammasome^13^ and regulates reactive oxygen species (ROS), further amplifying its protective effects against oxidative stress.^15^ Tissue damage in periodontitis is driven by the oxidative stress caused by neutrophil‐derived‐ROS, which induces lipid peroxidation, DNA damage, and protein oxidation.^16^ α‐Klotho is also an integral component of the fibroblast growth factor (FGF) receptor complexes, facilitating the binding of endocrine FGFs to their respective receptors. FGF‐2 plays a pivotal role in wound healing and the regeneration of periodontal tissues by promoting the differentiation of periodontal ligament cells into osteoblasts and cementoblasts, while also stimulating angiogenesis.^17^ As such, ⍺‐Klotho was found to reduce vascular rigidity and to prevent cardiac fibrosis, contributing to enhanced cardiovascular health^18^; to regulate phosphate and calcium homeostasis through its interaction with fibroblast growth factor‐23 (FGF23), and to reduce oxidative stress, inflammation, and fibrosis, thereby preserving kidney function.^19^ In the endocrine system, it improves insulin sensitivity and glucose metabolism, offering protection against metabolic syndrome and type 2 diabetes. It regulates lipid metabolism to prevent dyslipidemia.^20^ This mechanism of action may extend its anti‐inflammatory effect in the oral cavity, specifically by inhibiting the progression of periodontitis and promoting regeneration and wound healing.^21^

Since periodontitis is linked to systemic diseases, and low serum α‐Klotho levels are associated with these diseases, it is possible that low α‐Klotho levels might influence the development and/or severity of periodontitis. This suggests that α‐Klotho could be a valuable biomarker for the early detection of periodontitis. The single study on this topic showed that lower serum α‐Klotho levels were associated with more severe stages of periodontitis.^22^ However, due to overlaps of this biomarker and possible confounders (such as age, diabetes, CVD, and CKD), this study aimed to determine if there is a genuine association between ⍺‐Klotho levels and periodontitis severity, while controlling for these confounders. This was performed using data from three combined NHANES cycles. The classification of periodontitis stage, grade, and extent was conducted using the Application of the 2018 periodontal status Classification to Epidemiological Survey data (ACES) guidelines.^23^

## 2 MATERIALS AND METHODS

### 2.1 Study design and population

This cross‐sectional study adhered to the Strengthening the Reporting of Observational studies in Epidemiology (STROBE) guidelines. Data were extracted from the National Health and Nutrition Examination Survey (NHANES) database, specifically utilizing three combined cycles (2009–2010, 2011–2012, and 2013–2014). The NHANES data represent a weighted sample of 961 participants, extrapolating to a U.S. population of 40,965,619 individuals. All materials were obtained from the website (https://wwwn.cdc.gov/nchs/nhanes/).

### 2.2 Data collection

The NHANES database provided variables for analysis. Demographic variables included age (years), sex (male/female), ethnicity (Mexican American, Other Hispanic, Non‐Hispanic White, Non‐Hispanic Black, and Other), smoking status (never, former, or current), ratio of family income‐to‐poverty (continuous), health insurance coverage (yes/no), and frequency of dental floss/device use (days per week). Clinical variables included glycosylated hemoglobin (HbA1c) levels (%), presence of kidney disease (yes/no), presence of congestive heart failure (yes/no), and presence of coronary heart disease (yes/no). Periodontal variables included periodontitis stage (I, II, III, and IV), grade (A, B, and C), and extent (localized and generalized). Periodontitis classification was determined according to the 2018 ACES guidelines.^23^

#### 2.2.1 α‐Klotho measurement

Serum α‐Klotho levels were quantified using a commercially available enzyme‐linked immunosorbent assay (ELISA) kit, following the manufacturer's instructions. All samples were analyzed in duplicate, and the mean of the two measurements was used for subsequent statistical analyses. The distribution of α‐Klotho levels was assessed for normality using the Shapiro‐Wilk test and visual inspection of histograms and Q‐Q plots. Outliers were identified using the 1.5 × IQR rule and were Winsorized to the nearest non‐outlier value to minimize their impact on statistical analyses.

#### 2.2.2 Definition of periodontitis

Periodontitis was diagnosed based on either interdental clinical attachment loss (CAL) of at least 1 mm at two or more non‐adjacent teeth, or buccal/oral CAL of at least 3 mm along with a probing pocket depth (PPD) of more than 3 mm at two or more teeth. Staging was classified as follows: stage I (maximum interdental CAL of 1–2 mm), stage II (maximum interdental CAL of 3–4 mm without PPD ≥ 6 mm at two or more non‐adjacent teeth), stage III (maximum interdental CAL of 3–4 mm with PPD ≥ 6 mm at two or more non‐adjacent sites, or maximum interdental CAL ≥ 5 mm with at least 10 opposing pairs of natural teeth), and stage IV (maximum interdental CAL ≥ 5 mm with less than 10 opposing pairs of natural teeth).^24^ PPD measurements at the distal surfaces of second molars were excluded from staging. Due to data constraints, furcation involvement and vertical bone loss were not used as criteria for stage adjustment.

Grading was determined using indirect measures of disease progression, calculated through relative CAL according to the ACES framework. Direct evaluation of progression requires prior CAL or radiographic bone loss (BL) data from earlier examinations to assess changes over a 5‐year period. However, such data are rarely available in large epidemiological studies. Instead, progression is estimated indirectly by calculating the ratio of radiographic BL, represented as a percentage of root length at the most affected tooth, to the individual's age. When radiographic BL data are unavailable, relative CAL as a percentage of root length can be derived using the formula: relativeCAL(%)=(100×CAL[mm])/rootlength[mm].^23^ Grades were further adjusted using modifiers for self‐reported daily cigarette consumption. Missing values for smoking were supplemented using plasma cotinine concentrations. The diabetes category was based on examinations of plasma HbA1c levels. If self‐reported smoking, cotinine concentrations, and plasma measurements were unavailable, participants retained their original grade. Individuals who did not meet the diagnostic criteria for periodontitis were classified as non‐periodontitis cases. Smoking exposure was classified using serum cotinine (ng/mL). Participants with cotinine < 10 ng/mL were categorized as non‐smokers, while cotinine ≥ 10 ng/mL indicated active smoking and was further stratified into light (10– < 100) and heavy (≥ 100) exposure. Because established NHANES/CDC definitions characterize secondhand smoke exposure among non‐smokers as 0.05–10 ng/mL, individuals in this range were included in the non‐smoker reference category by design.^25^, ^26^

### 2.3 Statistical analysis

Statistical analyses were performed using SPSS version 26.0 (IBM, Armonk, NY, USA) and G*Power 3.1.9.7. An a priori power calculation indicated that n = 961 participants would provide 80% power to detect a minimum odds ratio of 1.2 at a two‐sided α = 0.05. This sample was randomly selected from the larger NHANES database. To ensure representativeness of the U.S. population, NHANES MEC examination weights were applied and adjusted as relative weights, maintaining the effective sample size. Initially, baseline associations between serum α‐Klotho and participant characteristics were assessed using two‐sample t‐tests (binary predictors) and Pearson’s correlation (continuous predictors). Periodontitis stage and grade were the primary ordinal outcome variables. Ordinal logistic regression models were used to evaluate associations between serum α‐Klotho levels and periodontitis outcomes, adjusting for confounders (age, sex, ethnicity, smoking status, HbA1c, comorbidities, socioeconomic status, and oral hygiene behaviors). The proportional odds assumption was verified. Secondary outcomes, such as the number of lost teeth, were analyzed using linear regression models, similarly adjusted for relevant confounders. Additionally, sensitivity analyses using binary logistic regression (stages I+II vs. III+IV and grades A+B vs. C) were performed. A significance level of α = 0.05 was used throughout, and all results were interpreted with consideration of clinical relevance rather than statistical significance alone.

## 3 RESULTS

### 3.1 Population and descriptive statistics

The study included a weighted sample of 961 patients from the NHANES database, representing a population of 40,965,619 individuals. The mean age of the participants was 48.7 ± 17.1 years. The sample comprised 51.2% females and 48.8% males. The prevalence of current smokers was 15.1%, while 29.6% were former smokers. The mean HbA1c level was 5.72% ± 0.88%, and the prevalence of kidney disease, congestive heart failure, and coronary heart disease was 1.8%, 2.3%, and 2.7%, respectively (Table 1 **)**.

**TABLE 1:** Association of Klotho and demographic and clinical profile: results of two‐sample *t*‐test and Pearson's correlation coefficient.

Characteristic	Value	*p* value	*Test Statistic*
Sample size	961 patients		
Age	Mean: 48.7 years (SD: 17.1 years)	0.002**	*r* = –0.10
Sex	Females: 51.2% Males: 48.8%	0.022*	*t* = 2.29
Smoking status	Current smokers: 15.1% Former smokers: 29.6% Never smokers: 55.3%	<0.001***	*t* = 3.91
HbA1c	Mean: 5.72 (±0.88)	0.044*	*r* = 0.07
Kidney disease prevalence	1.8%	0.082	t=1.74
Congestive heart failure prevalence	2.3%	0.080	t=1.75
Coronary heart disease prevalence	2.7%	0.421	t=0.80
Klotho protein level	Mean: 838.1 ± 275.5 pg/mL Median: 796.4 pg/mL (IQR: 658.3–969.1)		

* *p* < 0.05.

** *p* < 0.01.

*** Values *p* < 0.001.

The mean Klotho protein level was 838.1 ± 275.5 pg/mL, with a median of 796.4 pg/mL (IQR: 658.3–969.1). The distribution of Klotho levels was approximately normal, with no significant outliers (Figure S1).

### 3.2 Analysis of stage

**FIGURE 1:**
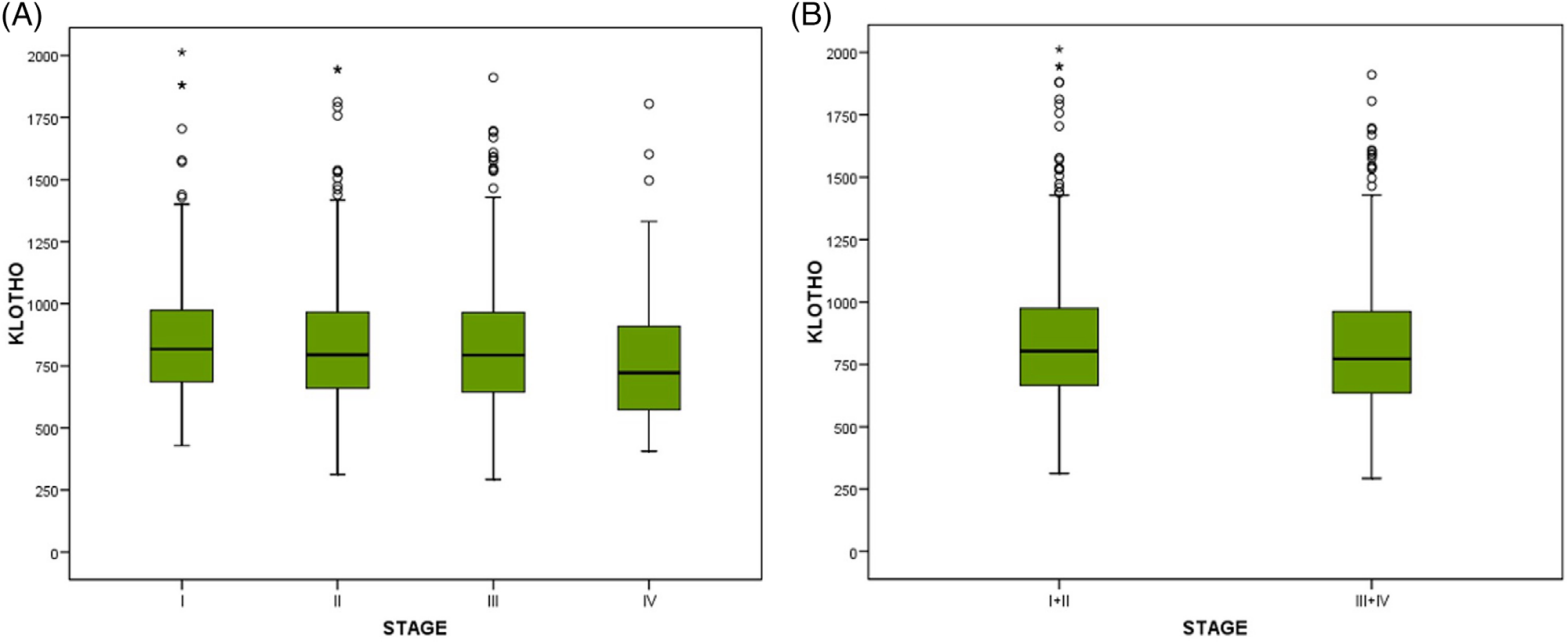
(A) Box plot shows a progressive reduction of the protein as the stage increased, but difference between the most severe stages was only 5.2%. (B) The probability of stage III+IV did not reduce significantly as the protein levels increased.

The primary outcome was the association between α‐Klotho levels and periodontitis stage (Table S1). Initial analysis revealed a progressive reduction in Klotho levels as the periodontitis stage increased, but the difference between the most extreme stages was only 5.2% (Figure 1A). Ordinal logistic regression revealed no significant association between α‐Klotho levels and the stage of periodontitis (OR = 0.9997, *p* = 0.116). After adjusting for potential confounders, the association remained non‐significant (OR = 1.0001, *p* = 0.547) (Table 2 **)**.

**TABLE 2:** Univariable and multivariable logistic regression results for staging (periodontitis severity was set as the dependent variable). Note that univariable analysis used a dichotomized smoking variable (ever vs never) as well as smoking frequency, whereas multivariable model used smoking status (never/former/current).

Univariable versus multivariable ordinal logistic regression results						
Variable/Category	Univariable analysis			Multivariable analysis		
	OR	95% CI	*p*‐value	OR	95% CI	*p*‐value
**Klotho**	0.9997	0.9992–1.0001	0.116	1.0001	0.9997–1.0006	0.947
**Sex** (Ref: Male)						
Female	0.43	0.34–0.55	<0.001***	0.48	0.34–0.58	<0.001***
**Age**	1.04	1.03–1.05	<0.001***	1.05	1.04–1.07	<0.001***
**Ethnicity** (Ref: Mexican American)						
Other Hispanic	0.88	0.42–1.74	0.660	0.69	0.31–1.50	0.348
Non‐Hispanic White	0.46	0.29–0.75	0.007**	0.39	0.22–0.69	0.001**
Non‐Hispanic Black	1.10	0.39–2.04	0.763	0.74	0.37–1.46	0.384
Other races	0.83	0.42–1.66	0.599	0.55	0.25–1.21	0.138
**Smoker** (Ref: Non‐smoker/No)						
Yes (Univariable)	2.28	1.78–2.90	<0.001***	–	–	–
Former (Multivariable)	–	–	–	1.68	1.25–2.25	0.001**
Current (Multivariable)	–	–	–	5.64	3.44–7.40	<0.001***
**Smoking frequency** (Ref: Every day)						
Some days	0.63	0.25–1.54	0.308	–	–	–
Not at all	0.34	0.23–0.51	<0.001***	–	–	–
**Number of cigarettes**	1.02	0.98–1.05	0.431	–	–	–
**Diabetes** (A1C)	1.38	1.21–1.57	<0.001***	1.12	0.97–1.30	0.129
**Kidney disease** (Ref: No)						
Yes	2.19	0.91–5.26	0.080	1.06	0.40–2.79	0.994
**Congestive heart failure** (Ref: No)						
Yes	3.19	1.46–6.93	0.004**	1.27	0.53–3.07	0.992
**Coronary heart disease** (Ref: No)						
Yes	3.72	1.80–7.67	<0.001***	2.28	1.03–5.08	0.043*
**Ratio family income‐to‐poverty**	0.76	0.70–0.82	<0.001***	0.85	0.78–0.94	0.001**
**Covered health insurance** (Ref: No)						
Yes	0.46	0.33–0.64	<0.001***	0.70	0.46–1.05	0.083
**Days usage dental floss/device**	0.98	0.94–1.02	0.390	–	–	–

*Note*: That univariable analysis used a dichotomized smoking variable (ever vs never) as well as smoking frequency, whereas multivariable model used smoking status (never/former/current).

Abbreviations: CI: Confidence intervals; OR, Odds ratio.

* *p* < 0.05.

** *p*<0.01.

*** *p* < 0.001.

For the covariates, age was positively associated with advanced stages (OR = 1.05, *p* < 0.001). Non‐Hispanic White patients had the lowest probability of advanced stages (OR = 0.39, *p* = 0.001). Smoking significantly increased the risk of advanced stages (OR = 5.04 for current smokers, *p* < 0.001). Coronary heart disease was also associated with higher stages (OR = 2.28, *p* = 0.043).

When stages were recorded into two categories (I+II vs. III+IV), α‐Klotho levels were not significantly associated with the advanced stages (OR = 0.9997, *p* = 0.267) (Figure 1B). After adjusting for confounders, the association remained insignificant (OR = 1.0002, *p* = 0.559). Age and smoking were positively associated with advanced stages (OR = 1.05, *p* < 0.001, and OR = 5.41, *p* < 0.001, respectively). Non‐Hispanic White patients had the lowest probability of advanced stages (OR = 0.37, *p* = 0.005).

### 3.3 Analysis of grade

**FIGURE 2:**
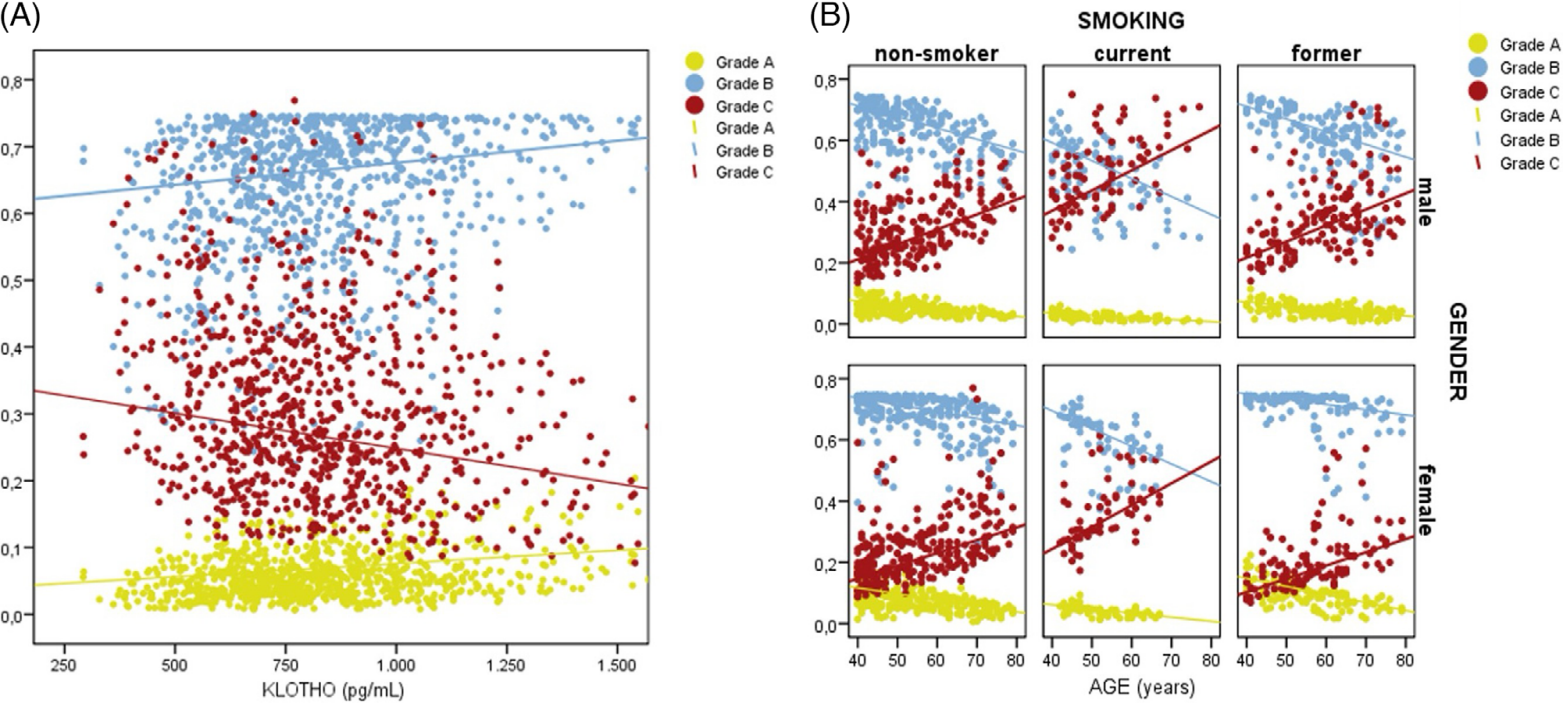
(A) Higher Klotho levels slightly increased the probability of earlier stages (I and II) and decreased advanced stages (III and IV), but the association was not significant (*p* = 0.547). (B) Estimated probability of advanced periodontitis stages (III and IV) by age, sex, and smoking status. Older age, male sex, and increased smoking intensity were associated with higher probabilities of advanced stages.

α‐Klotho levels were significantly associated with the grade of periodontitis in univariable analysis (OR = 0.9993, *p* = 0.008). However, after adjusting for confounders, the association was no longer significant (OR = 0.9996, *p* = 0.144) (Figure 2A; Table 3 **)**.

**TABLE 3:** Univariable and multivariable logistic regression results for grading (periodontitis severity was considered as the dependent variable).

Univariable versus multivariable ordinal logistic regression results						
Variable/Category	Univariable analysis			Multivariable analysis		
	OR	95% CI	*p*‐value	OR	95% CI	*p*‐value
Klotho	0.9993	0.9989–0.9998	0.008**	0.9996	0.9991–1.0001	0.144
Sex (Ref: Male)						
Female	0.58	0.44–0.75	<0.001***	0.60	0.45–0.81	0.001**
Age	1.02	1.01–1.04	0.001**	1.03	1.01–1.04	0.001**
Ethnicity (Ref: Mexican American)						
Other Hispanic	0.90	0.41–1.96	0.791	0.91	0.38–2.15	0.822
Non‐Hispanic White	0.61	0.36–1.02	0.060	0.61	0.33–1.13	0.115
Non‐Hispanic Black	0.77	0.39–1.52	0.457	0.63	0.29–1.33	0.223
Other races	1.42	0.68–2.99	0.353	1.12	0.48–2.64	0.788
Smoker (Ref: Non‐smoker)						
Yes (Univariable)	2.05	0.53–0.81	0.148	–	–	–
Former (Multivariable)	–	–	–	0.88	0.64–1.23	0.465
Current (Multivariable)	–	–	–	1.99	1.33–2.98	0.001**
Smoking frequency (Ref: Every day)						
Some days	1.21	0.93–1.57	0.158	–	–	–
Not at all	1.05	0.77–5.45	0.003**	–	–	–
Number of cigarettes	0.99	0.95–1.03	0.640	–	–	–
Diabetes (A1C)	1.27	1.09–1.47	0.001**	1.16	0.98–1.36	0.075
Kidney disease (Ref: No)						
Yes	3.73	1.44–9.72	0.007**	3.19	1.11–9.13	0.031*
Congestive heart failure (Ref: No)						
Yes	1.53	0.67–3.54	0.314	0.82	0.31–2.17	0.693
Coronary heart disease (Ref: No)						
Yes	1.66	0.77–3.60	0.197	1.24	0.52–2.95	0.628
Ratio family income‐to‐poverty	0.94	0.90–0.98	0.005**	0.92	0.83–1.02	0.101
Covered health insurance (Ref: No)						
Yes	0.72	0.50–1.04	0.077	1.07	0.69–1.69	0.741
Days usage dental floss/device	0.94	0.90–0.98	0.005**	0.96	0.91–1.01	0.102

*Note*: That univariable analysis used a dichotomized smoking variable (ever vs never) as well as smoking frequency, whereas multivariable model used smoking status (never/former/current).

Abbreviations: CI: Confidence intervals; OR, Odds ratio.

* *p* < 0.05.

** *p*<0.01.

*** *p* < 0.001.

Female patients had a lower probability of advanced grades compared with males (OR = 0.60, *p* = 0.001). Age was positively associated with advanced grades (OR = 1.03, *p* = 0.001). Current smokers had a higher risk of advanced grades (OR = 1.99, *p* = 0.001). Kidney disease was also associated with advanced grades (OR = 3.19, *p* = 0.031). When all three predictors of cigarette smoking, age and sex were combined, there was no significant difference noted in Klotho levels (Figure 2B).

**FIGURE 3:**
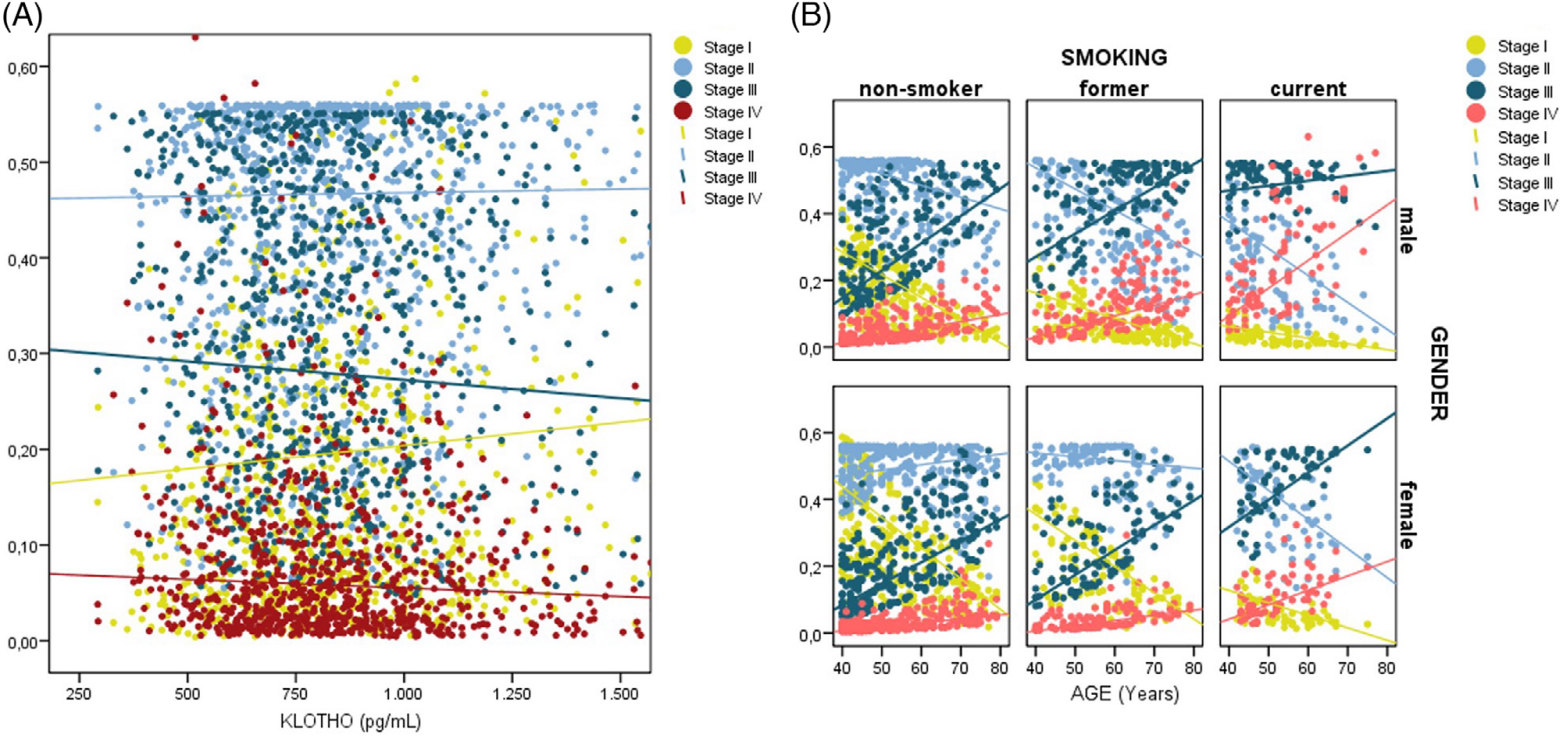
(A) The estimated probability of grade C decreased as the protein increased. (B) Combining all three predictors: sex, age, and smoking.

In the binary analysis of grade (A+B vs. C), α‐Klotho levels were significantly associated with grade C in univariable analysis (OR = 0.9993, *p* = 0.013). However, after adjusting for confounders, the association was no longer significant (OR = 0.9996, *p* = 0.172) (Figure 3A).

Female patients had a lower probability of grade C (OR = 0.61, *p* = 0.002). Current smokers had a higher risk of grade C (OR = 2.25, *p* < 0.001). Kidney disease was also associated with grade C (OR = 3.33, *p* = 0.026) (Figure 3B).

### 3.4 Analysis of the number of lost teeth

The mean number of lost teeth was 4.5 ± 5.4. α‐Klotho levels showed a weak negative correlation with the number of lost teeth (*r* = −0.07, *p* = 0.023) (Figure S2). However, after adjusting for confounders, the association was not significant (*p* = 0.413).

Older age, smoking, and coronary heart disease were associated with a higher number of lost teeth (*p* < 0.001). Patients with a higher income‐to‐poverty ratio and those who used dental floss regularly had fewer lost teeth (*p* < 0.001).

## 4 DISCUSSION

The principal aim of this study was to examine whether there is a robust relationship between serum α‐Klotho protein levels and the severity of periodontitis to explore α‐Klotho's potential as a biomarker or therapeutic target in periodontal disease. While preliminary univariable analysis suggested that α‐Klotho deficiency increased periodontitis severity, our adjusted multivariable regression analyses found no statistically significant association between serum α‐Klotho concentrations and the Stage or Grade of periodontitis, once confounding factors were controlled for. Similarly, although a weak inverse correlation was observed between α‐Klotho and tooth loss, this relationship was not sustained after multivariable adjustment. Age, smoking status, kidney disease, and coronary heart disease emerged as significant predictors of periodontitis severity and tooth loss, underscoring the multifactorial etiology of periodontal diseases. These findings align with previous studies identifying key demographic and clinical predictors of periodontitis severity, independent of serum α‐Klotho levels. Advancing age, smoking status, lower socioeconomic status, and certain comorbidities such as CVD and kidney disease were strongly associated with more severe periodontitis.^27^, ^28^

α‐Klotho is involved in anti‐inflammatory processes, antioxidant mechanisms, and the regulation of bone metabolism, all vital pathways in periodontal tissue destruction and regeneration.^13^, ^29^, ^30^ It mitigates oxidative stress by modulating ROS production and suppressing inflammatory mediators such as NF‐κB and the NLRP3 inflammasome.^13^, ^31^ Furthermore, α‐Klotho regulates calcium‐phosphorus metabolism and vitamin D homeostasis by interacting with FGF23, potentially influencing alveolar bone remodeling.^32^ This evidence highlights the importance of further investigation into α‐Klotho as a biomarker or therapeutic target, despite our study's lack of strong epidemiological associations.

A previous study^22^ reported a negative association between serum α‐Klotho levels and advanced stages of periodontitis using similar NHANES data from a single cycle. The discrepancy between our findings and those of Ni et al.^22^ can be attributed to several factors. First, differences in methodological approaches to defining periodontitis severity may have contributed to the divergent results. Ni et al.^22^ grouped stage I and II periodontitis together due to the low sample size of stage I, whereas our study used the comprehensive 2018 ACES guidelines for a much more accurate and nuanced classification of Stages and Grades. Although the 2018 ACES guidelines are widely accepted as a framework to date, their application is not without limitations. In particular, classification based on staging, grading, and extent may be prone to variability, given differences in clinical measurements, case definitions, and diagnostic thresholds across studies. These inherent challenges can introduce a degree of error in categorizing disease severity and may partly explain discrepancies observed between studies.

Second, our study combined three NHANES cycles and applied specific statistical weighting to achieve U.S. population representativeness, potentially leading to different population characteristics.^23^ Another notable methodological difference involves sample size and statistical power. Ni et al.^22^ used an overpowered sample size, which increased statistical power but may have identified statistically significant yet clinically irrelevant effects due to extremely small effect sizes. Our study, in contrast, adjusted the sample size to *n* = 961 based on a rigorous power calculation aimed at detecting a minimum clinically relevant difference of approximately 20%. By doing so, we sought to avoid spurious significance from trivial effect sizes, ensuring that significant findings were both statistically and clinically meaningful. This may partially explain why we found no statistically significant association between α‐Klotho levels and periodontitis severity. Lastly, the mentioned study did not explicitly mention using NHANES sample weighting in their statistical analyses. Given NHANES’ complex data structure, appropriate weighting is crucial for obtaining nationally representative estimates and providing more accurate population‐level inferences, which could account for the divergence in findings. Ni et al.^22^ modeled serum α‐Klotho as the outcome (dependent variable) and included periodontitis stage (I/II, III, IV) and other covariates as independent predictors in a stepwise multiple linear regression model, whereas in our study, periodontitis was considered the outcome, leading to the use of different statistical models and resulting in the inability to directly compare the studies.

Differences between the present findings and those reported by Ni et al. may, in part, be attributed to methodological and analytical distinctions between the two studies. Ni et al. utilized data from a single NHANES cohort (2013–2014) and identified a significant negative correlation between serum α‐Klotho levels and stage III/IV periodontitis, using a multiple linear (stepwise) regression model in which α‐Klotho served as the dependent variable and periodontitis stages were included among several independent predictors. Their multivariable model was adjusted for multiple covariates, including age, sex, race, BMI, smoking, alcohol intake, vitamin D, blood parameters, and systemic conditions such as diabetes, cardiovascular disease, and hyperlipidemia. Our statistical modeling was designed to adjust for important confounding factors such as age, sex, ethnicity, smoking status, HbA1c levels, kidney disease, congestive heart failure, coronary heart disease, socioeconomic factors, and oral hygiene habits. In epidemiological research, even minor variations in covariate choices can significantly impact observed associations. In contrast, the current study examined the reverse relationship, modeling periodontitis severity as the dependent variable and α‐Klotho as an independent predictor, using an ordinal logistic regression framework. These two analytical approaches rely on different statistical assumptions and outcome definitions, which likely contribute to the divergent results. Therefore, the inconsistency between studies may reflect not only differences in sample composition but also variation in the directionality of the models and statistical methodologies employed. To enable meaningful cross‐study comparison, future analyses should adopt consistent outcome measures and modeling strategies, ideally applying the same dependent variable across datasets.

However, our cross‐sectional study design presents limitations. First, the lack of longitudinal data prevents us from drawing causal conclusions about the role of α‐Klotho in periodontitis progression. Second, despite thorough adjustments for confounders, residual confounding from genetic predispositions, medication history, nutritional status, or differences in oral microbiome profiles might still exist. Third, our method of classifying periodontitis severity used clinical measures such as clinical attachment level, periodontal probing depth, and tooth loss, but did not distinguish between tooth loss due to periodontitis versus caries, possibly introducing classification bias. Lastly, while our patient selection ensured adequate power to detect clinically relevant effect sizes, it may have reduced sensitivity to detect subtle biological relationships at the population level.

To fully understand serum α‐Klotho in periodontal pathogenesis, future studies should include prospective and mechanistic research. Experimental studies could investigate molecular interactions between α‐Klotho and periodontal pathogens or inflammatory pathways. Longitudinal cohort studies with standardized periodontal assessments, detailed clinical histories, and precise control of confounding factors would be crucial for confirming the α‐Klotho role in periodontal disease progression. Moreover, for the purpose of comparability across studies, it is essential that future research adopts consistent variable definitions—specifically, evaluating periodontitis severity as the outcome and α‐Klotho as the independent variable, as done in the present study—to enable direct and meaningful cross‐study interpretation.

## 5 CONCLUSIONS

In conclusion, although no significant independent associations were found between serum α‐Klotho levels and periodontitis severity after adjusting for confounders, our findings affirm the complex nature of periodontitis pathogenesis. Demographic and clinical factors such as age, smoking, and comorbidities continue to be influential predictors of periodontal disease severity. Further research is warranted to explore the potential biological role of α‐Klotho in periodontal health and its systemic implications.

## AUTHOR CONTRIBUTIONS

**Sahar Baniameri**: Methodology; investigation; writing—original draft; writing—review and editing. **Hamoun Sabri**: Project administration; investigation; writing—original draft; writing—review and editing. **Pranav Kataria**: Data curation; data analysis, and Writing—original draft. **Hend Abulatifa**: Methodology; data curation, and writing—original draft. **Parham Hazrati**: Methodology; writing—review and editing. **Hom—Lay Wang**: Supervision; writing—review and editing. **Muhammad H. A. Saleh**: Conceptualization; supervision; methodology; writing—review and editing.

## CONFLICT OF INTEREST STATEMENT

The authors declare no conflicts of interest in this study.

## Supporting information



Supporting Information

## Data Availability

The datasets analyzed in this study are publicly available from the National Health and Nutrition Examination Survey (NHANES).
