# Supplementary material for: Association between serum α‐Klotho levels and severity of periodontitis in a representative U.S. population
Source: J Periodontol. 2026 Feb 28;97(6):1340–50. doi: 10.1002/jper.70080 (PMC13350410; doi:10.1002/jper.70080)
Supplement: Supplementary file 1 — Supporting Information [file JPER-97-1340-s001.docx]

**Supplementary Materials**

**Supplementary Figure 1.** Distribution of Klotho protein across the population. The box gathers 50% of cases, being divided by the median. Lower and upper box limits are the 1st and 3rd quartiles: Below them are 25% and 75% respectively of the sample. Vertical segments are extended until acceptable values, showing atypical as circled and extreme as asterisk symbols


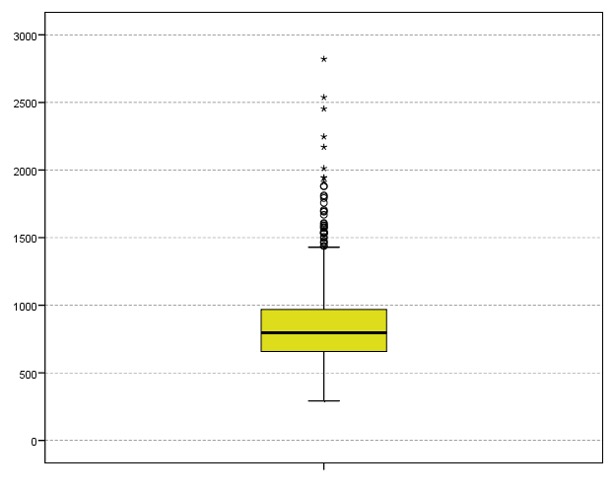


**Supplementary Figure 2.** Serum α-Klotho levels showed a weak negative correlation with tooth loss (r = -0.07, p = 0.023), which became non-significant after adjustment (p = 0.413). Older age, smoking, and coronary heart disease were linked to greater tooth loss, while higher income and regular flossing were associated with less tooth loss.


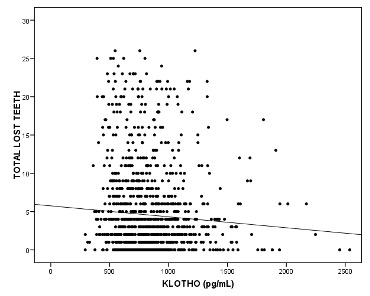


**Supplementary Table 1.** Klotho (pg/ml) levels by Periodontitis stage (weighted by MEC exam Relative weight)

|  | *Stage* | | | | |
| --- | --- | --- | --- | --- | --- |
|  | Total | I | I | III | IV |
| N | 961 | 185 | 450 | 268 | 58 |
| Mean | 838.1 | 863.6 | 837.6 | 830.7 | 795.0 |
| Standard Deviation | 275.5 | 250.2 | 281.5 | 275.9 | 302.3 |
| Minimum | 292.2 | 427.6 | 313.0 | 292.2 | 405.7 |
| Maximum | 2,820.9 | 2,012.8 | 2,820.9 | 1,910.8 | 1,805.2 |
| Percentile 25 | 658.3 | 685.3 | 659.6 | 647.2 | 583.7 |
| Median | 796.4 | 817.8 | 794.2 | 793.5 | 721.5 |
| Percentile 75 | 969.1 | 974.0 | 977.7 | 969.1 | 912.5 |
